# COVID‐19 restrictions increased perceptions of social isolation for older people discharged home after rehabilitation: A mixed‐methods study

**DOI:** 10.1111/ajag.13062

**Published:** 2022-03-10

**Authors:** Georgia Hogan, Nicholas F. Taylor, Leslie Robins, Michele L. Callisaya, Megan Snowdon, Chris Moran, David A. Snowdon

**Affiliations:** ^1^ Physiotherapy Department Peninsula Health Frankston Victoria Australia; ^2^ Allied Health Clinical Research Office Eastern Health Box Hill Victoria Australia; ^3^ School of Allied Health Human Services and Sport La Trobe University Melbourne Victoria Australia; ^4^ Peninsula Clinical School Central Clinical School Monash University Melbourne Victoria Australia; ^5^ Academic Unit Peninsula Health Frankston Victoria Australia; ^6^ National Centre for Healthy Ageing Monash University Melbourne Victoria Australia

**Keywords:** activities of daily living, community integration, community participation, COVID‐19, rehabilitation

## Abstract

**Objective:**

To explore older persons’ perceptions of the impact of COVID‐19 restrictions on participating in community activities after discharge from inpatient rehabilitation.

**Methods:**

Mixed‐methods study design. Participants were older adults who were discharged home following inpatient rehabilitation. Interviews were conducted with 70 participants, with a variety of diagnoses, 8 weeks after discharge from inpatient rehabilitation. Frequency of participation in domestic, leisure/work and outdoor activities was measured using the Frenchay Activities Index (FAI). Qualitative analysis was completed using qualitative content analysis and triangulated with FAI scores.

**Results:**

In all, 70 older adults (mean age: 73.0 years, SD: 9.9; 59% female) participated in the study. The overarching theme was that participants felt socially isolated following discharge from rehabilitation, with COVID‐19 restrictions increasing perceptions of social isolation and complicating their return to participating in community activities. The four categories informing the overarching theme were as follows: physical health was the primary limitation to participation in community activities; COVID‐19 restrictions limited participation in social activities and centre‐based physical rehabilitation; low uptake of videoconferencing to facilitate socialisation and rehabilitation; and reduced incidental physical activity. Mean FAI score was 21.2 (SD 7.8), indicating that participants were moderately active. Participants most commonly performed domestic activities (mean: 10.0, SD: 4.1), followed by outdoor activities (mean: 6.6, SD: 3.5) and leisure/work activities (mean: 4.5, SD: 2.5).

**Conclusions:**

COVID‐19 restrictions exacerbated perceptions of social isolation and the limitations already imposed by poor physical health after discharge from rehabilitation. The findings highlight the need for rehabilitation that addresses the psychological and social dimensions of community reintegration.

**Practice impact**

COVID‐19 restrictions exacerbated older peoples’ perceptions of social isolation and limitations already imposed by poor physical health on discharge from inpatient rehabilitation. Participants also reported limited access to rehabilitation and low uptake of videoconferencing technologies. Targeted strategies that facilitate socialisation and rehabilitation of older people may improve community reintegration following discharge from inpatient rehabilitation.

## 1 INTRODUCTION

Community integration is a focus of rehabilitation and involves physical, social and psychological dimensions.^1^ In the months following discharge from hospital, older people commonly report difficulty accessing the community, attending their prior social arrangements and lack of confidence with returning to their usual activities.^2^, ^3^ These difficulties returning to participation in society can adversely affect both quality of life and sense of belonging.^2^

In 2020, the COVID‐19 pandemic and social distancing measures changed the way we live and function in the community. In Melbourne, Australia, these measures included restrictions on the number of people who could gather, maintaining a distance of 1.5 metres between individuals, limits on the distance that people could travel from their place of residence, and the mandatory wearing of facemasks outside the home.^4^ When measures were most restrictive, people could only leave their home for shopping, essential work, exercise and health care/caregiving.^4^ These measures restricted people’s capacity to socialise and led to feelings of isolation, which negatively affected well‐being and mental health.^5^

The restrictions associated with the COVID‐19 pandemic may have affected how people participate in the community. More people have reported an interest in exercise during lockdown, and spent more time participating in moderate physical activity.^6^, ^7^ Digital communication technologies have had an increased presence in people’s lives, enabling socialisation with friends/family and working from home.^8^, ^9^ The latter resulted in less time spent commuting and improvements in work–life balance.^8^ Health care has rapidly embraced technology to improve the efficiency of health‐care delivery in response to demand created by the COVID‐19 pandemic.^10^

However, it is unclear how the COVID‐19 pandemic impacted on how older people discharged home from inpatient rehabilitation reintegrated into the community. Given this population already face difficulties reintegrating into the community, they may have been more susceptible to the negative impacts of the COVID‐19 pandemic.^2^, ^3^ Alternatively, digital communication technologies may have assisted them to overcome the physical and psychological impairments that commonly restrict them from participating in community activities.^2^, ^3^, ^9^ A better understanding of how the COVID‐19 pandemic impacted on their participation in community activities may inform future rehabilitation initiatives, and health care/social supports. Therefore, the aim of this study was to explore the perceived impact of COVID‐19 restrictions on participating in community activities after discharge from inpatient rehabilitation.

## 2 METHODS

### 2.1 Study design

A mixed‐methods design was used. We used qualitative research methods, including telephone interviews, to explore participant perceptions of the impact of COVID‐19 restrictions on participation in community activities.^11^ Concurrently, we also administered a quantitative descriptive survey of participation in activities of daily living, the Frenchay Activities Index (FAI), for the purpose of triangulation and to obtain a more comprehensive account of the phenomenon of interest.^12^ Participants provided written informed consent and the study received ethics approval from the Peninsula Health Human Research Ethics Committee (HREC) LNR/58068/PH‐2019.

### 2.2 Participants

Participants were recruited from two inpatient rehabilitation wards at a public health network located in metropolitan Melbourne, Australia that services over 300,000 people.^13^ Using a consecutive sampling method, all rehabilitation inpatients who were about to be discharged home from the two rehabilitation wards between February 2020 and July 2020 were screened to participate in the study. Rehabilitation inpatients who met the following inclusion criteria were eligible to participate in the study: adults aged ≥50 years discharging home from inpatient rehabilitation; pre‐morbidly able to walk independently with or without an assistive device; cognitive capacity to provide informed consent; able to walk independently or with only supervision, cueing/coaxing on discharge; and able to communicate in English. Inpatients discharging to residential care settings were excluded. Potential participants were identified at daily ward team meetings and inpatients who met the inclusion criteria were approached by a member of the research team, face‐to‐face prior to discharge home, to invite to participate in the study. All participants included in this study were exposed to social distancing measures following discharge from inpatient rehabilitation.

### 2.3 Interview and data items

Interviews were conducted at 8 weeks after discharge from inpatient rehabilitation. One researcher completed telephone interviews, typically of 20–30 min duration. The female research assistant was a qualified physiotherapist with 12 months experience of conducting telephone interviews. The interviewer had no prior relationship with the participants; however, participants were made aware of the interviewer’s role on the research team. The interview guide consisted of questions related to the impact of COVID‐19 restrictions on the participant’s capacity to complete their daily activities following discharge from rehabilitation (open‐ended questions) and walk in the community (closed‐ended question) (Table 1). The interview guide was pilot‐tested with two rehabilitation inpatients and no changes were made to the interview guide following pilot‐testing.

**TABLE 1:** Interview guide

Question Type	Question
Closed‐ended question (4‐point Likert scale)	How much has COVID‐19 restrictions limited your walking in the community? □ Large limitation □ Moderate limitation □ Small limitation □ No limitation
Open‐ended questions	How have COVID‐19 restrictions limited your ability to perform your usual daily activities, such as shopping; socialise with friends or family; walk outdoors/physical activity/exercise; pursue hobbies; gardening and household activities; participate in gainful work?
	Tell me how you have arranged any alternatives to complete your usual daily activities?
	Describe any other factors that have limited these activities and how have they limited them?
	Describe any ways in which your living arrangement changed since the introduction of COVID‐19 restrictions and if so, how have they changed?

Concurrently, the researcher also administered the FAI.^12^ The FAI measures the frequency of domestic, outdoor and leisure/work activities. It comprises 15 items each scored 0–3, which are summed to provide a total score (range: 0–45) and three sub‐scale scores (range: 0–15): domestic, outdoor and leisure/work activities.^12^ Higher scores indicate more frequent participation in activities. A score between 0 and 15 is classified as inactive, 16 and 30 moderately active, and 31 to 45 very active.^14^ The FAI has demonstrated the evidence of construct validity and is reliable in older patient groups.^12^

The following socio‐demographic variables were collected from participant medical records: diagnosis, age, sex, gait aid use at discharge, living arrangement and length of hospital stay.

### 2.4 Analysis

A researcher documented participant responses to interview questions at the time of the interview. Immediately following the interview, the researcher reviewed their documentation of the responses and kept a reflective diary of their own thoughts on the phenomena of interest. Participants were offered the opportunity to review the interviewers’ summary notes (i.e. their responses) but none accepted this invitation.

The interviewer’s summary notes were subjected to inductive qualitative content analysis.^11^ First, two researchers familiarised themselves with the data by reading and re‐reading the text. Then, researchers independently classified the text into meaning units (e.g. words, sentences and paragraphs related to each other through their content and context) using a software package (NVivo, Version 12; QSR International, Doncaster, Victoria, Australia). The meaning units were condensed and labelled with a code. The codes were then sorted into four categories through discussion with the two researchers and the interviewer. The researchers then re‐read the transcripts to confirm the categories and ensure no new categories arose. No new themes arose, suggesting that saturation had been achieved. Finally, the research team reflected on the categories and an overarching theme was formulated. Researchers established trustworthiness by means of triangulation with responses to the FAI (methodologic triangulation) and among researchers (investigator triangulation).^15^

Frenchay Activities Index data were described using means (standard deviation). Responses to the closed‐ended question on impact of COVID‐19 restrictions on the participant’s capacity to walk in the community were analysed descriptively by reporting the proportion of responses on the 4‐point Likert scale. Quantitative data analysis was completed using SPSS 26.0 (IBM Corp, Armonk, New York, USA).

## 3 RESULTS

### 3.1 Participant characteristics

A total of 70 rehabilitation inpatients were interviewed. In all, 42 rehabilitation inpatients refused to participate in the study. The majority of participants were female (*n* = 43, 59%) with an average age of 73 years (SD 9.9) (Table 2). In all, 16 (23%) were admitted to rehabilitation with a traumatic orthopaedic diagnosis, 19 (27%) an elective orthopaedic diagnosis, 10 (14%) a neurological diagnosis and 25 (36%) another diagnosis. No participants were diagnosed with COVID‐19. Participants were moderately active, with a mean FAI total score of 21.2 (SD 7.8). Participants most commonly performed domestic activities (mean 10.0, SD 4.1), followed by outdoor activities (mean 6.6, SD 3.5) and leisure/work activities (mean 4.5, SD 2.5). Forty‐seven per cent (*n* = 34) reported that COVID‐19 restrictions had ‘no limitation’ on their walking in the community, 24% (*n* = 17) reported a ‘small limitation’, 11% (*n* = 8) reported a ‘moderate limitation’ and 18% (*n* = 13) reported a ‘large limitation’.

**TABLE 2:** Socio‐demographic and clinical characteristics of patients included in the bivariate and multiple regression analyses

Variable	*n* = 70
Diagnosis, *n* (%)	
Orthopaedic trauma	16 (23)
Lower limb fracture	11 (16)
Upper limb fracture	2 (3)
Spine fracture	3 (4)
Orthopaedic elective	19 (27)
Total knee replacement	10 (14)
Total hip replacement	8 (11)
Hallux osteotomy	1 (2)
Neurological	10 (14)
Stroke	9 (13)
Spinal cord injury	1 (2)
Other	25 (36)
Falls	6 (9)
Deconditioned/functional decline	8 (11)
Respiratory	2 (3)
Abdominal surgery	3 (4)
Cardiac	4 (6)
Low back pain	1 (2)
Lower limb amputation	1 (2)
Age, *years*, mean (SD)	73.0 (9.9)
Sex: Female, n (%)	41 (59)
Gait aid use at discharge, n (%)	58 (83)
Living alone, n (%)	28 (40)
Length of hospital stay, *days*, mean (SD)	22.6 (14.8)
FAI total score, *units*, mean (SD)	21.2 (7.8)
FAI domestic score, *units*, mean (SD)	10.0 (4.1)
FAI leisure/work score, *units*, mean (SD)	4.5 (2.5)
FAI outdoor score, *units*, mean (SD)	6.6 (3.5)

FAI, Frenchay Activities Index.

### 3.2 Theme and categories

#### 3.2.1 Social isolation following discharge from inpatient rehabilitation

**FIGURE 1:**
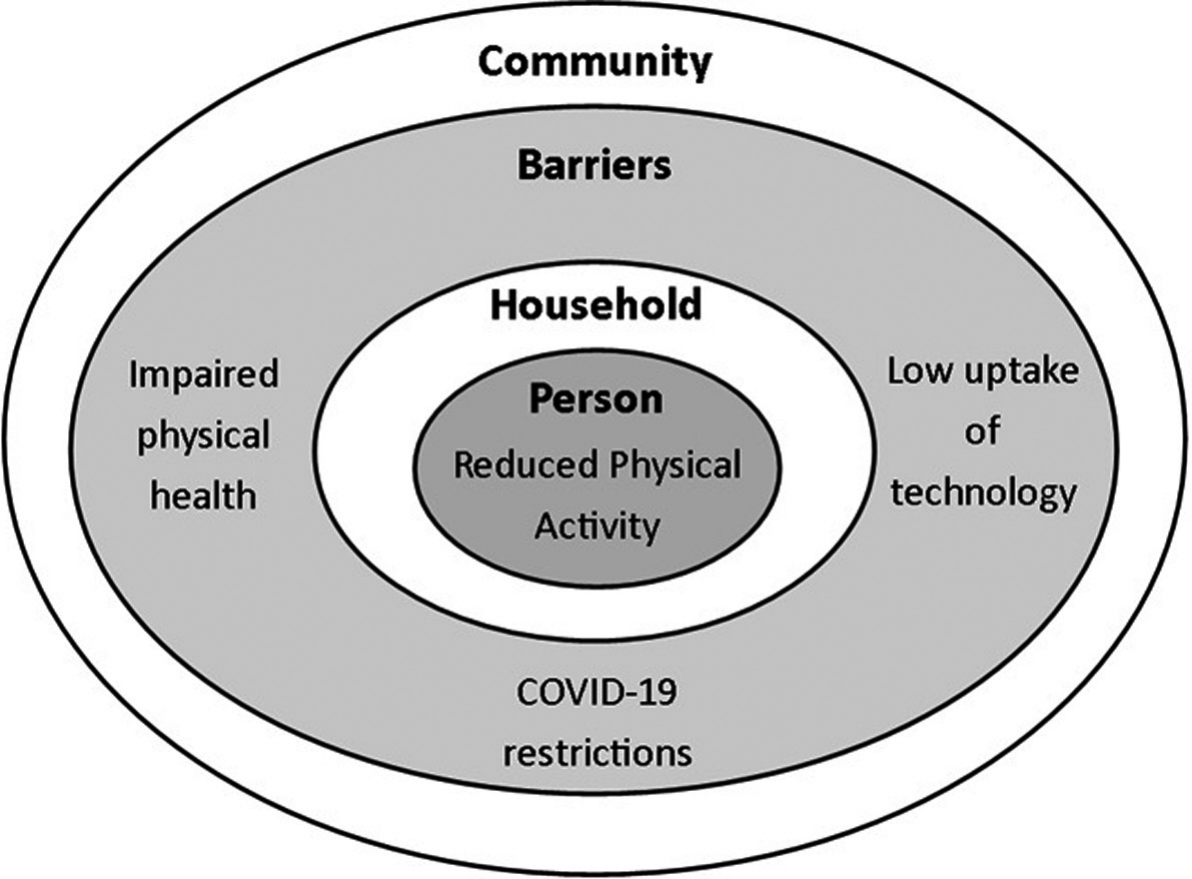
Social isolation following discharge from inpatient rehabilitation

The overarching theme was that participants expressed a feeling of social isolation following discharge from inpatient rehabilitation. Despite participants reporting their physical health was the primary limitation to participation in community activities, they recognised that COVID‐19 restrictions limited their socialisation with friends, families and other people in the community. They also reported that COVID‐19 limited access to rehabilitation and social support services, which increased their reliance on family. Participants reported limited use of technology to facilitate socialisation or rehabilitation, and little incentive to leave the home. This meant participants were mostly housebound and did little physical activity (Figure 1).

#### 3.2.2 Physical health was the primary limitation to participation in community activities

Participants reported impaired physical health was the main limitation to participating in their usual activities, including those completed in the community.

I have difficulty breathing which limits what I do. (P53)

This led to participants requiring assistance from family or friends to complete shopping or heavy household activities.

Doing the laundry and shopping has been hard since leaving hospital, so I get my daughter to wash and shop for me. (P67)

Only a few participants reported that they were reluctant to access the community due to a fear of COVID‐19. Participants emphasised that physically they were not likely to be accessing the community much more than if there were no restrictions.

I’m not limited by COVID‐19 as I would have stayed home to focus on my recovery. (P32)

#### 3.2.3 COVID‐19 restrictions limited participation in social activities and physical rehabilitation

Participants identified that COVID‐19 restrictions, in particular social distancing rules, had greatest impact on their participation in social activities. Not being able to see their family and friends in‐person took an emotional toll.

They (COVID‐19 restrictions) have immensely limited my socialisation with friends and I’m devastated that I cannot see them. (P64)

Participants who could physically access the community also reported limitations on their social activities. These participants reported that organised group activities such as ten pin bowling (P47), golf (P74), Mahjong card group (P22), choir (P58) and exercise group (P13) were cancelled due to restrictions. However, they did not report finding alternative arrangements (e.g. using technology) to navigate restrictions and facilitate socialisation.

Participants reported that they were restricted to independent walking or exercise programs, due to the cancellation of centre‐based physiotherapy. Participants reported that they missed the socialisation associated with attending these appointments in‐person. Instead of attending centre‐based appointments, participants commonly received a limited number of home‐visits and phone calls from community physiotherapists to monitor progress.

Physiotherapy has been cancelled, but they have given me an exercise program and call me to see how I am going. (P60)

While formal support services were available (e.g. shopping assistance, home cleaning services), participants reported the number of hours available was reduced due to restrictions. This led to reliance on family and friends for assistance.

Since my stroke a few years ago, I’ve had a carer who helps me to walk down the street, and do some shopping or go for a coffee. But because of COVID they are only visiting twice a week, so my wife has to help. (P8)

#### 3.2.4 Low uptake of videoconferencing to facilitate socialisation and rehabilitation

Participants rarely reported using videoconferencing to socialise with family and friends. Instead, they reported the telephone as their main method of communication, often citing a lack of skill in using technology.

I miss meeting my friends at the club and watching the horses, but I speak to them on the phone. (P16)

I’m not good with technology so I just use the phone. (P23)

Similarly, participants reported that physiotherapy and medical telehealth appointments were predominantly via telephone, with no participants reporting they used videoconferencing.

My GP calls me weekly. They prefer you don’t come into the practice. (P76)

#### 3.2.5 Reduced incidental physical activity

Participants reported the reduction in participation in community activities led to reduced physical activity. Participants only left the house to attend medical appointments, when these were scheduled face‐to‐face, and some reported leaving the house to complete shopping for basic grocery items when family were not available to assist.

I’m essentially locked in the house, except for medical appointments. (P35)

Few participants reported completing a daily walking program. However, their physical health and the perception they needed to conserve energy to complete essential activities, such as meal preparation, limited them to household walking.

I walk each day, but only to the letterbox so I have energy for household chores. (P64)

Participants who reported walking outdoors for the purpose of physical activity/exercise were active prior to their hospital admission, completed shopping, walked with their dog or were accompanied by their spouse.

There are the physiotherapy exercises that I do each day and walking with my wife. (P51)

The qualitative findings converged with FAI scores (Table 3).

**TABLE 3:** Triangulation of qualitative and quantitative findings

Qualitative finding	Convergence with quantitative finding
Participants socially isolated, with COVID‐19 restrictions increasing perceptions of social isolation	Converged with finding that participants scored lowest in the leisure/work subscale of the Frenchay Activities Index
Physical health was the primary limitation to participation in community activities, with few participants reporting a fear of COVID‐19 limiting their participation in community activities	Converged with finding that the majority (71%) of patients reported that COVID‐19 restrictions had no or small limitation on their walking in the community
Participants predominantly completed physical activity/exercise within the house and only left the house to complete shopping and attend medical appointments	Converged with finding that participants’ scores were low/moderate in the outdoor activities’ subscale of the Frenchay Activities Index
Physical health and COVID‐19 restrictions limited participation in community activities, and that participants had low levels of incidental activity, only leaving the house for shopping or medical appointments and conserving energy for household activities	Converged with finding that participants’ Frenchay Activities Index total score indicated they were moderately active. AND Converged with finding that participants’ scores were highest in the domestic subscale of the Frenchay Activities Index

## 4 DISCUSSION

COVID‐19 restrictions increased perceptions of social isolation of older people following discharge from inpatient rehabilitation. While participants perceived their physical health was the primary limitation to participating in community activities, they reported that COVID‐19 restrictions limited participation in social activities as well as access to rehabilitation and support services. In response to COVID‐19 restrictions, participants reported low uptake of videoconferencing technology and low levels of incidental activity.

Poor physical health limiting return to community activities is a significant issue for older people who return home after hospitalisation.^2^, ^3^ Many have conditions that are complex and chronic, which can lead to exacerbation of symptoms and rehospitalisation.^16^ As such, limitations to community participation can persist following discharge home and have an adverse effect on a sense of belonging.^2^ A holistic approach to rehabilitation is required, which includes development of skills to self‐manage their condition in addition to traditional physical rehabilitation.^17^

Social isolation was a major issue for participants following discharge home.^5^ Social distancing measures likely exacerbated an issue that was already present, as feelings of isolation and difficulty participating in social activities has been identified as one of the primary difficulties when reintegrating into the community following discharge from hospital.^2^, ^3^ Similarly, prior to the pandemic, it was common for older people to report an increased reliance on family and friends for assistance with domestic and community activities.^2^, ^3^ Given that participants in our study reported a reduction in the availability of services to assist with these activities, it is likely the burden on family and friends was greater.^18^ These findings indicate that social distancing measures may have worsened the psychological impact of returning home following hospitalisation, for both patients and their families.

Community reintegration may have been further complicated by the limited availability of face‐to‐face community rehabilitation services. Participants were limited to home exercise programs, with telephone follow‐up, as centre‐based rehabilitation was stopped due to COVID‐19 restrictions. For community‐dwelling older people, home‐based programs can be equally as effective as centre‐based programs for improving physical outcomes.^19^ However, they lack personal interactions that may address feelings of social isolation.^20^ Furthermore, home‐based programs often require a level of supervision from professionals, and without supervision patient adherence can be low, particularly in people with multiple co‐morbidities.^19^, ^21^ Therefore, the lack of centre‐based rehabilitation services may have been detrimental to the rehabilitation of some people.

Videoconferencing may facilitate supervision of home exercise programs and monitoring of symptoms during exercise more than telephone.^21^ However, no participant in our study reported using videoconferencing. Older people are 76% less likely to use video visits (i.e. telehealth via video) than younger adults.^22^ Possible reasons for low uptake are a lack of skill in the use of smartphones or computers, lack of access to technology, the high cost of technology and inappropriate size of technology (e.g. smartphones).^23^, ^24^ Another possible reason for low uptake could be that videoconferencing was not offered to participants. Therapists may not have access to technology to facilitate videoconferencing, they may lack skill in use of videoconferencing or they may perceive any technical issues that arise as a deterrent to using videoconferencing.^25^ To address these barriers, both patients and therapists likely require support and education in the use of new technology.^26^

Physical limitations and restrictions to socialising and face‐to‐face rehabilitation appeared to limit participants’ incidental activity, with participants mostly limited to light domestic activities. In the short term, reductions in physical activity may lead to poorer mental health and well‐being.^27^ In the long term, there could be adverse effects for physical health and cognition.^28^, ^29^, ^30^ Prior to the pandemic, physical activity was already below recommended levels in people following serious medical events such as stroke and hip fracture.^29^, ^30^ The pandemic may have reduced these levels even further, which could have long‐lasting adverse effects on their health.

Our study highlights the difficulty of reintegrating older adults into the community following hospitalisation and has important implications for practice. Difficulty returning to social activities and feelings of isolation post‐hospitalisation were a significant issue for participants in our study and highlight the need for rehabilitation models of care to include components that address all dimensions of community reintegration rather than just the physical dimension.^1^, ^31^ While poor physical health was identified as a major contributing factor to limited participation in community activities, the COVID‐19 pandemic further complicated community reintegration through the introduction of social distancing measures. However, the pandemic also provides an opportunity to better utilise communication technology to deliver health care. Participants in our study reported limited use of communication technology (i.e. videoconferencing software) to facilitate rehabilitation and social activities. Education and resources on the use of communication technologies could help to increase the uptake of these technologies, by older people and therapists, to facilitate community reintegration following hospitalisation.^26^ These resources could be useful during the COVID‐19 pandemic and also following the pandemic for older people who are socially isolated and/or have limited access to rehabilitation services, such as those living remotely, those with impairments that limit their travel, and those who are immunocompromised. Policies supporting the provision of telehealth, such as the recent extension of funding for telehealth services through the Medicare Benefits Schedule,^32^ should also provide incentive for health providers to increase the use of communication technologies in their clinical practice.

### 4.1 Study limitations

This study is the first we are aware of to explore perceptions of the impact of COVID‐19 restrictions on participating in community activities after discharge from inpatient rehabilitation. There was a relatively large sample (*n* = 70) increasing confidence that we achieved data saturation; and participants were a diverse range of patients with various diagnoses, which increases the generalisability of our findings. We also used a mixed‐methods approach to achieve a more comprehensive account of the phenomenon of interest. A limitation is that we did not audio‐record patient interviews. Audio or visual recordings of participant interviews are more likely to accurately reflect participant views than researcher notes. To address this limitation, the interviewer took notes during and immediately after the interview and kept a reflective diary. We also performed content analysis with a low level of abstraction and interpretation, as demonstrated by our findings being close to the text (i.e. example quotes).^33^ Another limitation is that we only recruited participants from one Australian rehabilitation centre, who were previously community ambulant and able to walk with only supervision, cueing/coaxing on discharge. This limits the generalisability of our results to higher functioning patients and public health settings that are similar to those in Australia.

## 5 CONCLUSIONS

Following discharge from inpatient rehabilitation, older people reported COVID‐19 restrictions adversely affected their ability to socialise, attend face‐to‐face rehabilitation and receive formal services. They reported that this led to an increased reliance on family and friends to provide informal care, and reduced activities outside of the home environment. These findings indicate that COVID‐19 restrictions exacerbated the limitations already imposed by poor physical health in this population. Our findings highlight that there is a need for holistic rehabilitation models of care that address the psychological and social dimensions of community reintegration in addition to the physical dimension. Further research investigating the effect of holistic rehabilitation models of care on community reintegration and the use of technology to provide this care is required.

## ACKNOWLEDGEMENTS

Open access publishing facilitated by Monash University, as part of the Wiley ‐ Monash University agreement via the Council of Australian University Librarians.

## CONFLICTS OF INTEREST

Chris Moran serves on the Australasian Journal on Ageing management committee.

## Data Availability

Data collected and analysed in this study are available from the authors on request.
